# Treatment of Milk Abscess

**Published:** 1854-08

**Authors:** 


*Treatment of Milk Abscess.*—We alluded, in terms of strong commendation, in a former report, to the plan of tonic medication in all cases of milk abscess, which is adopted by Mr. Paget at St. Bartholomew's Hospital.

Very numerous cases of this affection, in all forms and stages, excepting perhaps the peracute, come under treatment in the out-patients' rooms of that Institution. Their subjects are generally cachectic women. Mr. Paget always opens the abscess as soon as fluctuation can be felt, by an incision as small as possible, scrupulously abstains from squeezing, and orders a small poultice to be applied over the parts where the skin is inflamed. Over the poultice a layer of cotton-wool is placed, and the whole slung to the neck in a handkerchief. A draught, containing quinine and the sulphate of iron, is ordered to be taken three times daily. Mr. Paget states, that, for many years, he has never, in a single case, had any trouble with fistulæ. We observe a great difference in practice among Hospital Surgeons as to making more than a mere puncture. On the whole, the latter mode appears to be the more successful; the constitutional treatment is doubtless, however, of the most importance.—*London Medical Times.*

